# Proaggregant nuclear factor(s) trigger rapid formation of α-synuclein aggregates in apoptotic neurons

**DOI:** 10.1007/s00401-016-1542-4

**Published:** 2016-02-02

**Authors:** Peizhou Jiang, Ming Gan, Shu-Hui Yen, Simon Moussaud, Pamela J. McLean, Dennis W. Dickson

**Affiliations:** Neuropathology Laboratory, Department of Neuroscience, Mayo Clinic, 4500 San Pablo Road, Jacksonville, FL 32224 USA

**Keywords:** Aggregation, Apoptosis, 6OHDA, Lamin B1, MPP+, Nuclear membrane integrity, Parkinson’s disease, Proaggregant nuclear factors, α-Synuclein

## Abstract

Cell-to-cell transmission of α-synuclein (αS) aggregates has been proposed to be responsible for progressive αS pathology in Parkinson disease (PD) and related disorders, including dementia with Lewy bodies. In support of this concept, a growing body of in vitro and in vivo experimental evidence shows that exogenously introduced αS aggregates can spread into surrounding cells and trigger PD-like pathology. It remains to be determined what factor(s) lead to initiation of αS aggregation that is capable of seeding subsequent propagation. In this study we demonstrate that filamentous αS aggregates form in neurons in response to apoptosis induced by staurosporine or other toxins-6-hydroxy-dopamine and 1-methyl-4-phenylpyridinium (MPP+). Interaction between αS and proaggregant nuclear factor(s) is associated with disruption of nuclear envelope integrity. Knocking down a key nuclear envelop constituent protein, lamin B1, enhances αS aggregation. Moreover, in vitro and in vivo experimental models demonstrate that aggregates released upon cell breakdown can be taken up by surrounding cells. Accordingly, we suggest that at least some αS aggregation might be related to neuronal apoptosis or loss of nuclear membrane integrity, exposing cytosolic α-synuclein to proaggregant nuclear factors. These findings provide new clues to the pathogenesis of PD and related disorders that can lead to novel treatments of these disorders. Specifically, finding ways to limit the effects of apoptosis on αS aggregation, deposition, local uptake and subsequent propagation might significantly impact progression of disease.

**Electronic supplementary material:**

The online version of this article (doi:10.1007/s00401-016-1542-4) contains supplementary material, which is available to authorized users.

## Introduction

Neuronal intracellular aggregates of α-synuclein (αS) in Lewy bodies and Lewy neurites are pathological features of Parkinson’s disease (PD) and related synucleinopathies such as dementia with Lewy bodies [1]. Although the mechanisms underlying αS pathology remain unclear, the possibility that abnormal conformers of αS may propagate from one cell to another has drawn increasing attention [2, 3]. Cell-to-cell propagation has been proposed to explain the spread of αS pathology observed in cross-sectional studies of brains with a range of severity of PD pathology and conceptually organized into stages by Braak et al. [4]. The presence of αS pathology in neurons of embryonic mesencephalic grafts implanted into the neostriatum to treat PD patients after a lag of about 10 years also supports the possibility that αS spreads from host to graft [5, 6]. In addition, a growing body of in vivo and in vitro experimental evidence supports the possibility that αS can spread from cell to cell [7–11]. Inherent to this hypothesis is initial formation of “seeds” of abnormal αS in selectively vulnerable neuronal populations, which subsequently propagate [3, 12]. The origin of the seeds in experimental studies has been abnormal conformers of recombinant wild-type or mutant αS or sonicated fractions “preformed fibrils” from post-mortem PD brains [8–10].

Neurodegeneration in PD and related disorders is a chronic, progressive process associated with neuronal cell death, associated energy failure and disruption of cellular integrity, which are features of cells undergoing apoptosis [13]. Evidence from postmortem brains of PD [14, 15], animal models of PD [16, 17], as well as in vitro cell culture studies [18, 19] strongly suggest that αS-associated neuronal death is apoptotic. Moreover, there is evidence for a proapoptotic environment in the substantia nigra in PD [14]. The identification of high-molecular-weight αS species in cells expressing αS subjected to apoptosis [20, 21] prompted us to investigate if αS aggregation is induced by apoptosis and to determine factor(s) that promote pathologic αS aggregation, which may be seeds for eventual cell-to-cell transmission.

In the present study, we employed cell- and animal-based studies to explore mechanisms of αS aggregation in apoptotic neurons. We demonstrate that filamentous αS aggregates rapidly form in apoptotic neurons through an interaction between αS and proaggregant nuclear factor(s) following disruption of the nuclear envelope. Moreover, αS aggregates released upon cell breakdown can be taken up by surrounding cells in both in vitro and in vivo experimental models. These data support the possibility that loss of nuclear membrane integrity plays an important role in αS pathology.

## Materials and methods

### Cell culture and maintenance

We used a dopaminergic neuronal cell model, BE(2)-M17D/3D5, which is derived from human neuroblastoma BE(2)-M17D cell line [22] that expresses human wild-type αS upon TetOff induction and displays neuronal phenotypes under retinoic acid-induced differentiation [23]. BE(2)-M17D/3D5 cells were maintained in DMEM/10 % fetal bovine serum with 2 μg⁄ml Tet at 37 °C and 5 % CO2. Cells were seeded at the density of 3 × 10^6^ cells/plate (100 × 20 mm, BD Biosciences, San Jose, CA) or 5.0 × 10^5^ cells/well in 6-well plates for biochemical analysis, and 2 × 10^4^ cells/well on coverslips in 24-well plates for immunocytochemistry. For differentiation and αS induction, media were replaced with Neurobasal medium (Invitrogen, Thermo Fisher, Waltham, MA), 2 % B-27 supplement (Invitrogen, Thermo Fisher), 2 mM l-glutamine (Sigma-Aldrich, Saint Louis, MO) and 10 μM RA (Sigma-Aldrich).

A previously described H4 neuroglioma cell line that inducibly co-expresses the N-terminal half of Venus YFP tagged to αS (V1S) and C-terminal half of Venus YFP tagged αS (SV2) upon TetOff induction, named as H4/V1S-SV2 [24], were maintained in OPTI-MEM (Invitrogen) medium supplemented with 10 % fetal bovine serum (Invitrogen), 200 µg/ml Hygromycin B, 200 µg/ml G418 and 2 μg⁄ml Tet and incubated at 37 °C.

The Lund human mesencephalic (LUHMES) cell line (LUHMES ATCC^®^ CR-2927) is a subclone of the Tet-controlled, v-myc-overexpressing human mesencephalic-derived cell line. It can be differentiated into dopamine-like neurons [25]. Cells were cultured in advanced DMEM/F-12/Glutamax medium supplemented with N2 supplement (Invitrogen) and 40 ng/ml FGF2 (R&D Systems, Minneapolis, MN) at 37 °C according to published methods [26]. Culture plates were pre-coated with 50 µg/ml poly-l-ornithine (Sigma-Aldrich) and 1 µg/ml fibronectin (Sigma-Aldrich, F1141). For differentiation, media were replaced with advanced DMEM/F-12/Glutamax/N2 medium (Invitrogen) containing 2 ng/ml human recombinant glial cell-derived neurotrophic factor (R&D Systems), 1 mM cAMP (Sigma-Aldrich) and 1 µg/ml Tet for 4 days.

For primary cultures, cortical/hippocampal neurons from embryonic C57BL/6 wild-type mice were seeded on poly-d-lysine (Sigma-Aldrich) coated 6-well plate at about 1 × 10^6^ cells per well. Experimental protocols for primary cultures were the same as those reported previously [27].

### Lentiviral plasmids and virus preparation

Lentiviral plasmids carrying αS (EX-G0543-Lv105) were purchased from Genecopoeia, Rockville, MD, and shRNAs of human LMNB1 were purchased from Sigma-Aldrich. The preparation of Lentivirus carrying genes of interest or shRNA was the same as described previously [28].

### Isolation of nuclear and cytoplasmic fractions

Nuclear and cytoplasmic fractions were isolated using NE-PER nuclear and cytoplasmic extraction reagents (Thermo Scientific, Waltham, MA) following the manufacturer’s instructions, but with some modifications. Briefly, cells were resuspended in cytoplasmic extraction reagent I, incubated for 10 min, followed by the addition of cytoplasmic extraction reagent II and centrifugation at 16000×*g* for 5 min. Supernatant was kept as cytoplasmic fraction. The insoluble pellet was further mixed with nuclear extraction reagent and subjected to sonication for 3 min followed by centrifugation at 16000×*g* for 10 min. The supernatant was then kept as nuclear fraction. The whole process was done on ice or at 4 °C. The bicinchoninic acid (BCA) assay was used for protein quantitation.

### Isolation of apoptotic bodies

Apoptotic bodies were isolated according to a previously reported protocol [29]. Medium from 10 plates (100 × 20 mm) of apoptotic neurons was collected and clarified from dead cells and cell debris by centrifugation (800×*g*, 10 min). The apoptotic body-enriched supernatant was further centrifuged (16000×*g*, 20 min) to generate a final pellet containing apoptotic bodies. The pellet was either stored at −80 °C for future use or resuspended immediately in PBS.

### Fractionation studies of apoptotic bodies and electron microscopy

Apoptotic bodies were fractionated to derive 1 % sarkosyl-insoluble material following previously reported protocols [27]. The sarkosyl-insoluble material was resuspended in 50 mM Tris (pH 8.0) and used for electron microscopy and immunoelectron microscopy as described previously [30].

### Sample preparation and western blot analysis

Cell cultures were harvested and centrifuged at 200×*g* for 15 min. The pellets were resuspended in MES buffer (20 mM MES, pH 6.8; 80 mM NaCl, 1 mM MgCl_2_, 2 mM EGTA, 10 mM NaH_2_PO_4_, 20 mM NaF, phenylmethylsulfonyl fluoride PMSF, 1 μg/ml and leupeptin, 10 μg/mL) [22] supplemented with phosphatase inhibitors and then sonicated for 1 min, followed by centrifugation at 180×*g* for 15 min. The whole process was done at 4 °C. The cell lysates were mixed with 6 × SDS-PAGE sample buffer (375 mM Tris–HCl, 12 % SDS, 60 % Glycerol, 12 % 2–Mercaptoethanol, 0.03 % Bromophenol blue), boiled for 10 min and resolved by SDS-PAGE using 10–20 % Tris/Glycine gel (Bio-Rad, Hercules, California). Precision Plus protein standards (Bio-Rad) were included as references. After gel electrophoresis, proteins were transferred onto polyvinylidene difluoride (PVDF) membranes. Antibodies used for western blot studies are as follows: total αS (Syn1; mouse monoclonal IgG1; cat. #: 610787) from BD Bioscience; phospho-serine-129 αS (pSyn #64, mouse IgG1; cat #: 015-25191) from Wako USA, Richmond, VA; pore membrane protein of 121 kDa (POM121) (N2N3, rabbit polyclonal; cat #: GTX102128) from GeneTex, Irvine, CA; lamin B1 (LMNB1) (rabbit polyclonal; cat #: 12987-1-AP) from Proteintech, Rosemont, IL; Histone H3 (rabbit polyclonal; cat # ab1791) from Abcam, Cambridge, MA; cleaved caspase 3 (rabbit polyclonal to human cleaved caspase 3 (Asp175); cat #: 9661) from Cell Signaling, Danvers, MA; α-tubulin (rabbit monoclonal; Epitomics cat #: 1878-1) from Abcam, Cambridge, MA; and β-actin (mouse monoclonal IgG2a; cat #: A5316) from Sigma, Saint Louis, MO (A5316). Western Lightning Plus ECL (PerkinElmer, Bridgeville, PA) or ECL™ Prime Western Blotting Detection Reagent (Fisher Scientific, Pittsburgh, PA) was used for visualization of protein immunoreactivities. The results of western blots were quantified using ImageJ software. Expression levels of proteins of interest were normalized to internal control. Data from at least 3 sets of independent experiments were analyzed by one-way ANOVA with Dunnett’s post hoc test for statistical significance.

### Time-lapse confocal microscopy imaging

H4/V1S-SV2 cells with αS induction for 5 days were cultured in reduced serum medium (Cat. No. 31985-062, Invitrogen) in Lab-Tek™ Chambered Cover Glass System (4 well, Nunc™ Lab-Tek II, Sigma-Aldrich). After exposure to staurosporine (STS), cells were subjected to time lapse imaging (interval time = 10 min, 16 h for early or 36 h for later stage of apoptosis) by confocal microscopy (Zeiss LSM 510, Carl Zeiss MicroImaging, Pleasanton, CA) at 37 °C to monitor formation and distribution of αS aggregates. Three independent experiments were performed to confirm the results. In each experiment, 5 fields (upper left, upper right, center, lower left and lower right) with at least 90 cells were chosen for counting the ratio of cells having predominant αS aggregation in nuclei after 16 h of STS treatment.

### Immunocytochemistry

Cells grown on cover slips were rinsed with PBS, fixed in 4 % paraformaldehyde and permeabilized with 0.1 M Tris-buffered saline (TBS; pH 7.6) containing 0.5 % Triton X-100 for 5 min. They were subsequently blocked with 3 % goat serum in TBS, incubated with primary antibodies (rabbit anti-Flag from Sigma Aldrich, mouse anti-Myc from cell signaling, or LB509 from Invitrogen) in TBS containing 1 % goat serum overnight at 4 °C and then incubated for 1 h with secondary antibodies. Immunolabeled cells were stained with nuclear stain DAPI (Invitrogen) for 10 min and observed by confocal fluorescence microscopy (Zeiss LSM 510, Carl Zeiss MicroImaging, Pleasanton, CA). Three independent experiments were performed to confirm the results. For each in situ cell uptake experiment, at least 60 αS-Flag cells were included for counting of cells bearing particles positive with Myc and Flag tags.

### Stereotaxic surgery

All animal procedures were approved by the Mayo Clinic Institutional Animal Care and Use Committee. C57BL/6 mice (12 months of age) were anesthetized with 3 % isoflurane and stereotaxically injected with different samples. For cell lysates and apoptotic bodies, 20 µg total protein per brain was injected. Control mice were injected with PBS to exclude any effects due to surgery and injection. A single-needle insertion (coordinates: *X* = 2.0 mm; *Y* = 0.2 mm; *Z* = 0.8 and 2.6 mm, respectively) into the left forebrain was used to deliver the inoculum to somatosensory cortex and dorsal neostriatum. Material was injected via a Hamilton syringe at a rate of 0.5 μl per min (5 μl total per site). After recovery from surgery, animals were monitored regularly. A week later, animals were subjected to transcardial perfusion with PBS, and brains were fixed in 10 % formalin followed by paraffin embedding. Coronal sections were cut at 5-micron thickness for immunohistochemical and immunofluorescent staining.

### Thioflavin T binding

Apoptotic bodies were resuspended in TBS at pH 7.4 and 10 µM Thioflavin T. Fluorescence was measured immediately at 440 nm (excitation)/460–600 nm (emission) with a Cary Eclipse fluorescence spectrophotometer (Varian, Palo Alto, CA). After subtraction of background signals from reagent alone, the fluorescent signals from the peak areas were integrated [31].

### Immunohistochemical and immunofluorescent staining

Sections of paraffin-embedded tissue were sequentially subjected to deparaffinization, rehydration, steaming in DAKO target retrieval solution pH 6.1 for 30 min, digestion with Protease 24 for 8 min at room temperature (only for staining with LB509) and blocking with Protein Block (X0909, DAKO, Carpinteria, CA) for 1 h at room temperature. For immunoperoxidase labeling, sections were treated with 3 % hydrogen peroxide to block endogenous peroxidase and then incubated with 5 % normal goat serum for 20 min to reduce non-specific labeling. Tissue sections were incubated with antibodies to αS (LB509, 1:100, Invitrogen) for 45 min and Envision-Plus labeled polymer HRP, rabbit or mouse (DAKO) for 30 min. Peroxidase labeling was visualized with a solution containing 3, 3′-diaminobenzidine (DAB-Plus). The sections were subsequently counterstained with Lerner 1 hematoxylin (14-930-11, Fisher Scientific) and coverslipped with cytoseal mounting medium (8310-16, Richard-Allan Scientific, Kalamazoo, MI). For immunofluorescence, sections were incubated with Protein Block (X0909, DAKO) for 1 h at room temperature. The sections were incubated with antibodies to MAP2 (1:1000, chicken polyclonal, EnCor Biotechnology, Gainesville, FL) and αS (LB509, 1:100, Invitrogen) at 4 °C overnight, followed by 1.5 h of incubation with secondary antibodies (1:500) after washing. Non-specific fluorescence signals were blocked by staining with Sudan Black. Sections were coverslipped with Vectashield mounting media (H-1200, Vector Laboratories, Burlingame, CA) and viewed with confocal microscopy.

## Results

### Induction of insoluble αS in cellular models by apoptosis

**Fig. 1:**
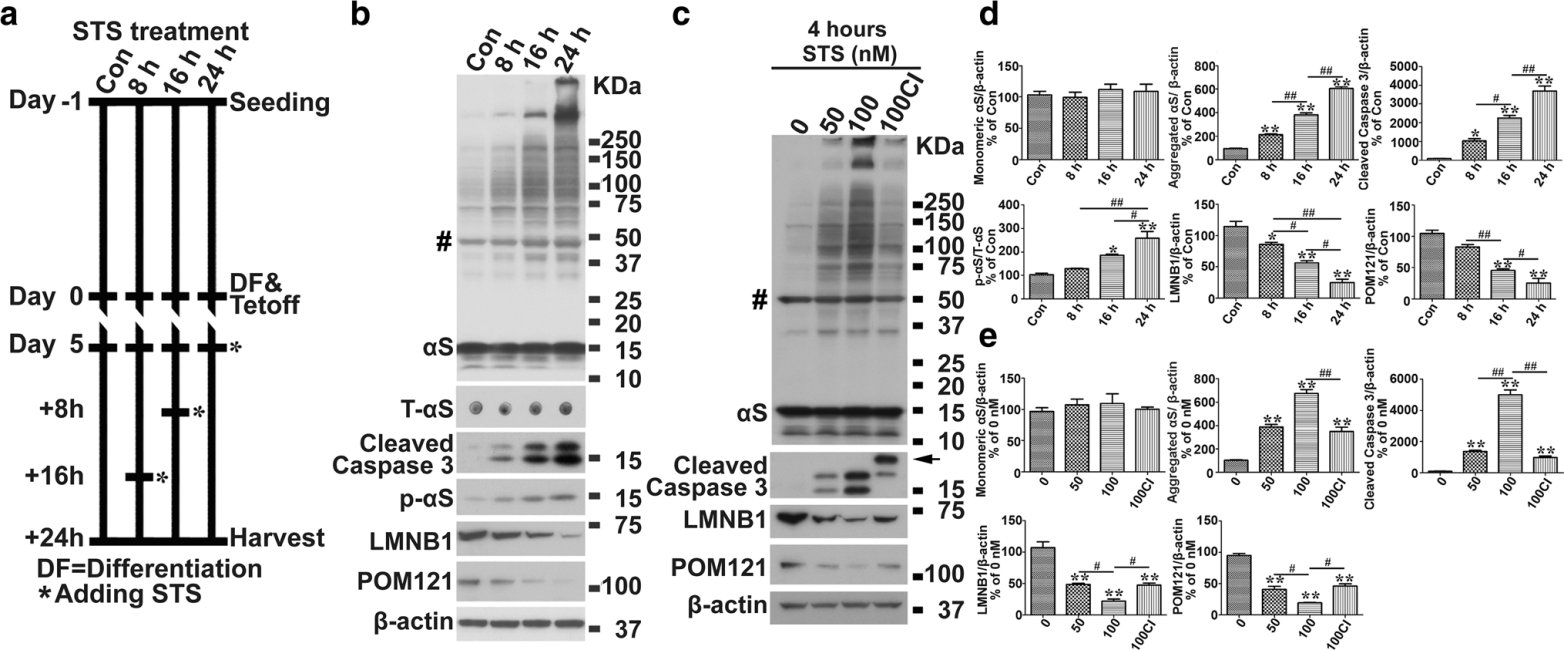
Apoptosis induces αS aggregation and phosphorylation in BE(2)-M17D/3D5 cells. **a** The basic paradigm for cell differentiation, αS induction and staurosporine (STS) treatment. BE(2)-M17D/3D5 cells with 5 days of differentiation and αS induction were exposed to 25 nM STS for 0, 8, 16 and 24 h before harvest. **b** STS treatment of BE(2)-M17D/3D5 cells induces apoptosis, αS aggregation and Serine 129 phosphorylation, and degradation of lamin B1 (LMNB1) and nuclear pore membrane protein 121 KDa (POM121) in a time-dependent manner. **c** High dose of STS treatment can rapidly trigger apoptosis and other aforementioned effects which can be significantly prevented by caspase inhibitor. BE(2)-M17D/3D5 cells with 5 days of differentiation and αS induction were exposed to 50 and 100 nM for 4 h before harvest. A group of 100 nM STS-treated sibling cultures was pretreated with caspase inhibitor (CI). All cell lysates were subjected to SDS-PAGE followed by western blotting with antibodies to interesting proteins. Dot blotting of cell lysates was used to detect total αS level (T-αS) for quantification of p-αS/T-αS immunoreactivities. Molecular weight standards were included as references. *Arrow* denotes a specific cleaved caspase 3 band in BE(2)-M17D/3D5 cells pretreated with caspase inhibitor. Number sign (*#*) denotes a non-specific Syn1-immunoreactive band on western blots of lysates from BE(2)-M17D/3D5 cells. **d**, **e** Statistical analysis of immunoreactivity of various proteins shown in (**b** and **c**). *Bar graphs* in **d** and **e** summarize the results of quantitative analyses of immunoreactivities of various proteins in cell lysates from three independent experiments represented by **b** and **c** with normalization against β-actin or T-αS immunoreactivities. The average values of control group (Con or 0 nM) were set as 100 %. *Error bars* represent standard error of the mean (*p < 0.05, **p < 0.01, comparing to control; #*p* < 0.05, ##*p* < 0.01, comparing subsets linked by *line*, *n* = 3)

To determine if apoptosis can trigger intracellular αS aggregation, staurosporine (STS), a well-known neuronal apoptosis inducer, was used to treat differentiated BE(2)-M17D/3D5 cells because it has been shown that exposure of neurons to 30–100 nM STS can induce concentration-dependent apoptotic degeneration [32]. As expected, BE(2)-M17D/3D5 cells exposed to 50 or 100 nM STS showed time- and dose-dependent increases in both cleaved caspase 3, a molecular marker of apoptosis, and in SDS-resistant aggregated αS (Fig. 1). αS aggregation was observed as early as 4 h after exposure to STS (Fig. 1c, e), indicating that factor(s) associated with apoptosis rapidly trigger pathologic assembly of αS. Pretreatment with a caspase inhibitor (CI)-Z-VAD-FMK, a cell-permeant pan-caspase inhibitor that irreversibly binds to the catalytic site of caspase proteases, significantly reduced both caspase activation and αS aggregation (Fig. 1c, e), confirming that apoptosis contributes to formation of αS aggregates.

**Fig. 2:**
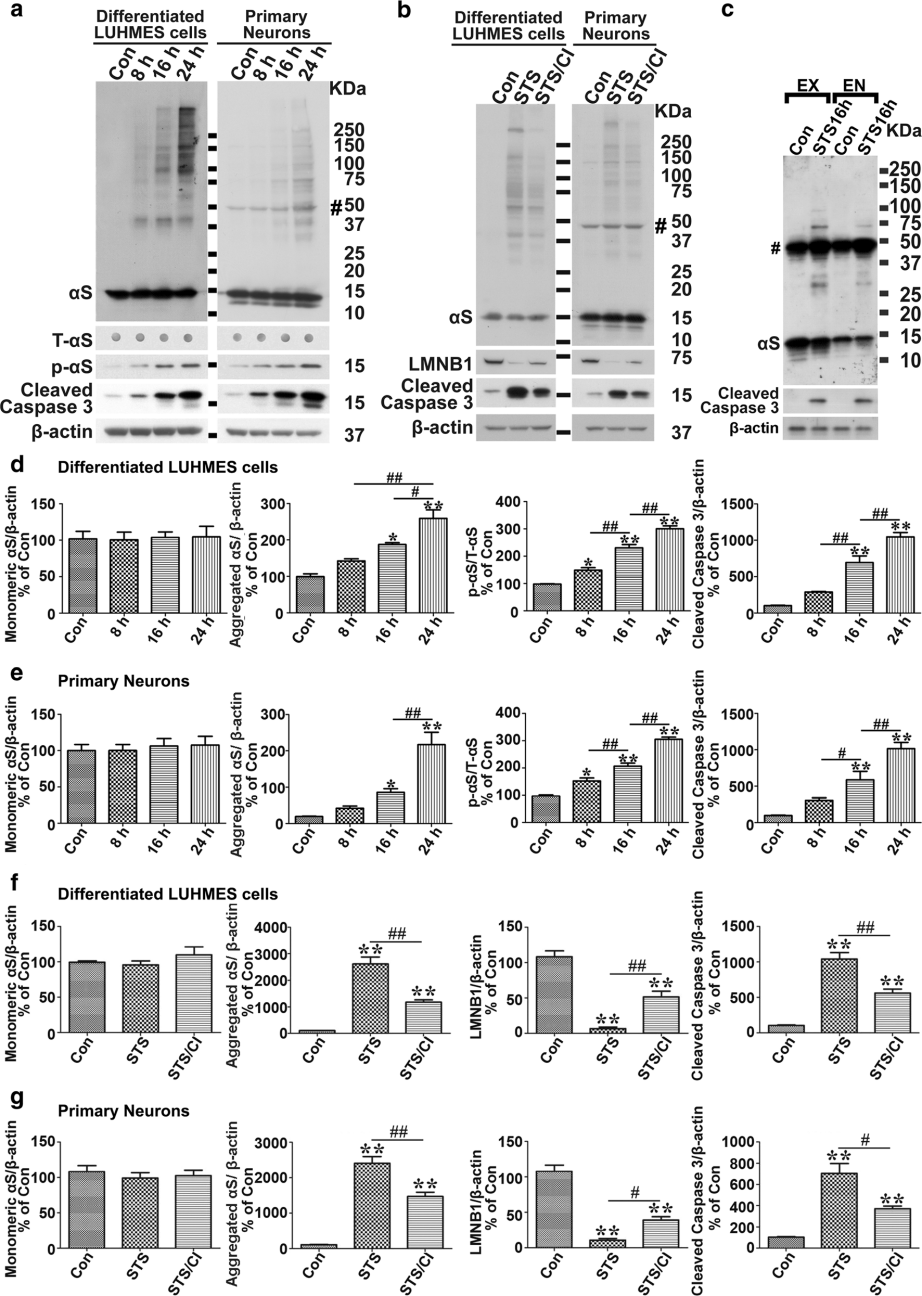
Apoptosis induces αS aggregation and phosphorylation in differentiated LUHMES and primary neurons. **a** STS treatment induces apoptosis, αS aggregation and phosphorylation in a time-dependent manner in differentiated LUHMES cells and primary neurons. Differentiated LUHMES cells and primary neuronal cultures overexpressing αS via infection with lentivirus carrying αS were, respectively, exposed to 100 nM STS for 0, 8, 16 and 24 h before being harvested. **b** Caspase inhibitor (CI) prevents STS-induced apoptosis and αS aggregation in differentiated LUHMES cells and primary neuronal cultures. Cultures without STS treatment were used as control (Con). STS-treated sibling cultures were pretreated with caspase inhibitor (STS/CI) to inhibit apoptosis. **c** Apoptosis induces aggregation of endogenous αS in mouse primary cultures. Primary neuronal cultures with only endogenously expressed αS (EN) or with exogenously overexpression of αS (EX) via infection with lentivirus carrying αS were exposed to 100 nM STS or vehicle (Con) for 16 h before harvest. All cell lysates were subjected to SDS-PAGE followed by western blotting with antibodies to proteins of interesting. Dot blotting of cell lysates was used to detect the total αS level (T-αS) for quantification of p-αS/T-αS immunoreactivities. Molecular weight standards were included as references. Number sign (*#*) denotes a non-specific Syn1-immunoreactive band on western blots of lysates from primary cultures. **d**–**g** Statistical analyses of immunoreactivities of various proteins shown in **a** and **b**. *Bar graphs* in **d**–**g** summarize results of quantitative analyses of immunoreactivities of various proteins in cell lysates from three independent experiments represented by **a** *left*, **a** *right*, **b** *left* and **b** *right* with normalization against β-actin or T-αS immunoreactivities. The average values of control group (Con) were set as 100 %. *Error bars* represent standard error of the mean (*p < 0.05, **p < 0.01, comparing to control; #*p* < 0.05, ##*p* < 0.01, comparing subsets linked by *line*, *n* = 3)

To determine whether αS aggregation in response to apoptosis is unique to BE(2)-M17D/3D5 cells, we included two other cell models, differentiated LUHMES cells (Fig. 2a) and primary mouse cortical neurons (Fig. 2b). Since increased levels of αS can accelerate aggregation [33], the two cell models were infected with lentivirus carrying human αS to increase its expression. In both cell models SDS-resistant αS aggregates formed after STS treatment in a time-dependent manner, and pretreatment with the caspase inhibitor blocked both apoptosis and αS aggregation (Fig. 2).

To further determine if apoptosis-triggered αS aggregation has physiological relevance, we used STS to treat primary neurons bearing only endogenous αS. As a positive control, we included sibling cultures infected with lentivirus carrying human αS. The results showed that endogenous αS (EN) can also form aggregates in response to STS treatment, but the magnitude of aggregation is much less compared to that in neurons overexpressing αS (EX) with lentivirus infection (Fig. 2c).

### Fluorescent complementation shows αS aggregates in nuclei during induction of apoptosis

**Fig. 3:**
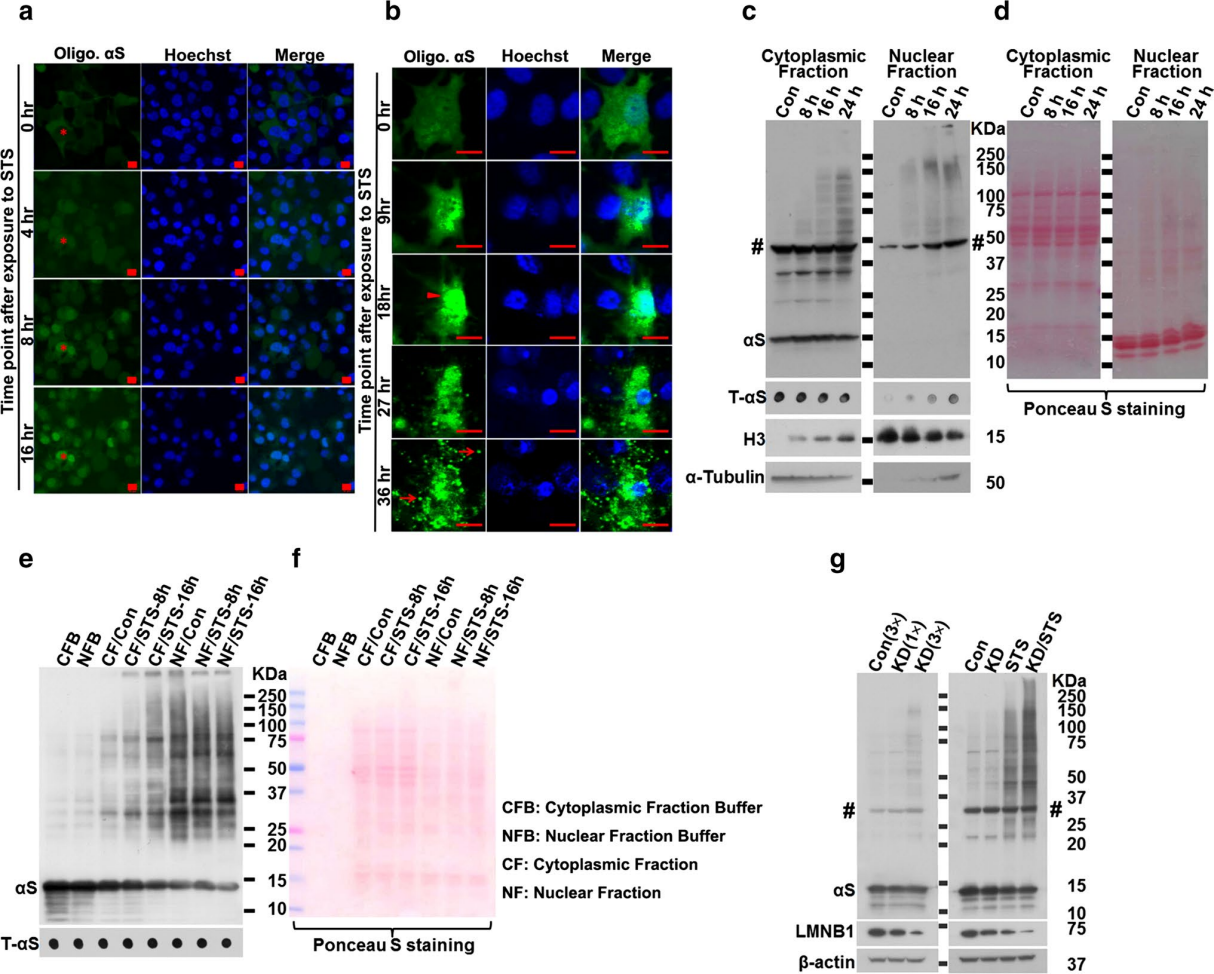
Disruption of nuclear envelope plays an essential role in the rapid αS aggregation during neuronal apoptosis. **a**, **b** αS aggregation in response to apoptosis induction predominantly occurred in nuclei rather than cytoplasm in live H4/V1S-SV2 cells. H4/V1S-SV2 cells cultured in Nunc™ Lab-Tek chambered cover glass system were induced to express H4/V1S-SV2 for 4 days, then maintained in media containing STS and Hoechst 33342 and subjected to time-lapse imaging using confocal fluorescence microscopy. A low magnification of live cell imaging demonstrates that about 94 % cells (30–32) had oligomeric αS (Olig. αS) predominantly in nuclei of cells (see *red asterisk* * in **a**) under 16 h of STS treatment. High magnification of live imaging of a single cell showed that the majority of αS aggregates appears first in nuclei (*arrow head* in **b** at 18 h) and then distributed to cytoplasm before finally being released to surrounding cells due to cell breakdown (*arrows* in **b** at 36 h). *Scar bar*: 10 µm. **c**, **d** Fractionation of cytoplasmic and nuclear proteins revealed the dynamic distribution of αS aggregates and other proteins in apoptotic neurons. Differentiated BE(2)-M17D/3D5 cells with 5 days of αS induction were, respectively, exposed to 25 nM for 0, 8, 16 and 24 h before harvest. **c** Nuclear and cytoplasm fractions were isolated and subjected to SDS-PAGE followed by western blotting. Dot blots were used to detect total αS (T-αS) for evaluation of the dynamic changes of T-αS in different fractions. α-Tubulin and histone H3 were probed to confirm there was no contamination between cytoplasmic and nuclear fraction in non-treated control group. **d** Ponceau S staining was performed before immunostaining to demonstrate total protein in each group. **e**, **f** Proaggregant nuclear factor(s) diffuse into cytoplasm with progression of apoptosis. Nuclear and cytoplasm fractions from null M17D neuroblastoma cells with 0, 8 and 16 h of STS treatment were, respectively, isolated. Fresh recombinant αS (10 µg) was incubated with the same amount of protein from each fraction for 30 min at 37 °C. The same amount of recombinant αS incubated with only cytoplasmic fraction buffer (CFB) or nuclear fraction buffer (NFB) was used as control. **e** Samples were subjected to SDS-PAGE followed by western blotting with monoclonal antibody to αS (Syn1). Dot blotting was used to confirm the comparable level of input recombinant αS in each sample. **f** Ponceau S staining was performed to demonstrate comparable levels of protein in all samples of cytoplasmic and nuclear fractions. **g** Disintegration of nuclear envelope is associated with rapid αS aggregation in neuronal cells in response to apoptosis induction. Differentiated BE(2)-M17D/3D5 cells with 5 days of αS induction infected with lentivirus carrying shRNA of LMNB1 and control vector were exposed to media with or without 25 nM STS for 16 h. Lentivirus infection was performed on the day 3 of culture. *Left*: one arbitrary unit (1x) or 3 units (3x) of lentivirus were applied to cells to obtain different extent of LMNB1 knockdown (KD). For control (Con), 3 units (3x) of lentivirus was used. *Right*: one arbitrary unit of lentivirus was used for both control (Con) and LMNB1 knockdown (KD). Cell lysates were subjected to SDS-PAGE and western blotting. *Number sign* (*#*) denotes a non-specific Syn1-immunoreactive band on western blots of lysates from BE(2)-M17D/3D5 cells in (**c**, **g**)

To study dynamic changes in αS aggregation during apoptosis, a fluorescence complementation assay with the H4/V1S-SV2 cell line was used. This cell line expresses αS tagged with a split Venus YFP molecule. Binding of V1S to SV2 upon αS–αS interaction can reconstitute fluorescence and indicates αS aggregation [24]. After 5 days of induction, H4/V1S-SV2 cells were exposed to 25 nM STS and subjected to time-lapse confocal microscopy to monitor αS aggregation. Unexpectedly, after 16 h of STS treatment, most cells (91 ± 3 %) showed predominant fluorescent signals in nuclei, suggesting that the nuclear environment in neurons undergoing apoptosis favors αS aggregation. At later stages of apoptosis, associated with nuclear condensation, αS aggregates were detected in the cytoplasm, and finally they were released into the media, where some bound to nearby cells (Fig. 3a, b).

### Apoptosis-induced αS aggregates are found in nuclear and cytoplasmic fractions

To support findings from live cell imaging, we performed nuclear and cytoplasmic protein fractionation of STS-treated neuronal cultures. The BE(2)-M17D/3D5 human dopaminergic cell line was chosen because it inducibly expresses wild-type human αS at a high level amenable to biochemical analyses [23]. Differentiated BE(2)-M17D/3D5 cells with 5 days of αS induction were collected at various time points following exposure to STS. Nuclear and cytoplasmic fractions were prepared and analyzed with dot blots, as well as with sodium dodecyl sulfate polyacrylamide gel electrophoresis (SDS-PAGE) western blots. As expected, dot blots revealed more αS in cytoplasmic than nuclear fractions (Fig. 3c, Supplementary Fig. A1), consistent with previous study results showing that αS is predominantly a cytoplasmic protein. Total αS progressively increased in the nuclear fraction following induction of apoptosis with STS, suggesting that αS translocates to the nucleus with apoptosis. Western blots showed that the cytoplasmic fraction had much more monomeric αS than the nuclear fraction when the same amount of total proteins from each fraction were compared; whereas higher molecular weight αS aggregates are more apparent in nuclear compared to cytoplasmic fractions (Fig. 3c, Supplementary Fig. A1). These results suggest that the nuclear fraction contains factor(s) that promote αS assembly. Moreover, αS aggregation increases in a time-dependent manner after induction of apoptosis in both nuclear and cytoplasmic fractions (Fig. 3c, Supplementary Fig. A1). Since translocation of αS to the nucleus persisted during apoptosis, it was not surprising to see progressive increase of αS aggregation in the nuclear fraction with progression of apoptosis. It was not immediately clear why aggregation of cytoplasmic αS also increases with progression of apoptosis; however, it is possible that nuclear factor(s) promoting aggregation may translocate from nucleus into the cytoplasm given that a representative nuclear protein (histone H3, Fig. 3c) gradually increases in cytoplasmic fractions with progression of apoptosis (Supplementary Fig. A1).

### Nuclear factor(s) that promote αS assembly shift to cytoplasm with apoptosis

To confirm the role of nuclear factor(s) in facilitating αS aggregation, the same amount of protein (50 µg) from nuclear and cytoplasmic fractions of differentiated null M17D cells (without αS overexpression) with or without 8 and 16 h of STS treatment was incubated with freshly prepared recombinant αS (10 µg) as described previously [30] for 30 min at 37 °C. At baseline, αS aggregates were minimal-to-nonexistent when recombinant αS was mixed with either cytoplasmic fraction buffer or nuclear fraction buffer, but αS aggregates rapidly appeared in preparations exposed to nuclear fractions from both STS-treated and non-treated cells (Fig. 3e, Supplementary Fig. A1), supporting the presence of nuclear factor(s) that facilitate αS aggregation. Interestingly, in STS-treated cells, the effect of cytoplasmic fraction on αS aggregation was positively correlated with the duration of treatment, consistent with the hypothesis that as apoptosis progresses, proaggregant nuclear factor(s) increasingly gain access to cytoplasm.

### Disruption of nuclear envelope integrity during apoptosis and αS aggregation

Given that nuclear envelopes block free passage of molecules between nucleus and cytoplasm and that apoptosis is associated with degradation of the nuclear envelope [34], we hypothesized that progressive increases in nuclear proaggregant factor(s) in the cytoplasm would correlate with disruption of the nuclear envelope. We addressed this by measuring the status of two key proteins of the nuclear envelope, pore membrane protein of 121 kDa (POM121) and lamin B1 (LMNB1), in STS-treated neuronal cultures for their relation to increases in αS aggregation (Fig. 1b, c).

We postulated that neurons with compromised nuclear envelope integrity due to decreased expression of key structural components of nuclear membrane would have increased susceptibility to STS and that αS aggregation would be increased in such cells. To test whether compromised nuclear envelope can trigger αS aggregation, neurons were subjected to knockdown of LMNB1 using lentivirus delivered shRNA, followed by STS or vehicle treatment. We found neurons deficient in LMNB1 (expressing less than 20 % of control levels) had significantly more αS aggregates than cultures exposed to the same dose of lentivirus carrying control vector (Fig. 3g, Supplementary Fig. A1), suggesting that loss of nuclear envelope integrity was associated with increased vulnerability to αS aggregation. Cultures with LMNB1 knockdown to around 50 % of control had similar expression of αS as controls, but they had significantly more αS aggregates than controls at the same dose and duration of STS treatment, suggesting that neurons with deficient constituent proteins of nuclear envelope are more vulnerable to αS aggregation.

### Phospho-serine 129 in αS aggregates of apoptotic neurons

Because phosphorylated αS (p-αS) accumulates in Lewy bodies and Lewy-related pathology [35], we immunostained apoptosis-induced αS aggregates for the presence of this post-translational modification. Western blots of cell lysates from differentiated BE(2)-M17D/3D5 cells, as well as LUHMES cells and primary neurons subjected to STS-induced apoptosis all had time-dependent increases of p-αS (Figs. 1b, d, 2a, d, e).

### Aggregation and phosphorylation of αS in neuronal cultures exposed to other neurotoxins

**Fig. 4:**
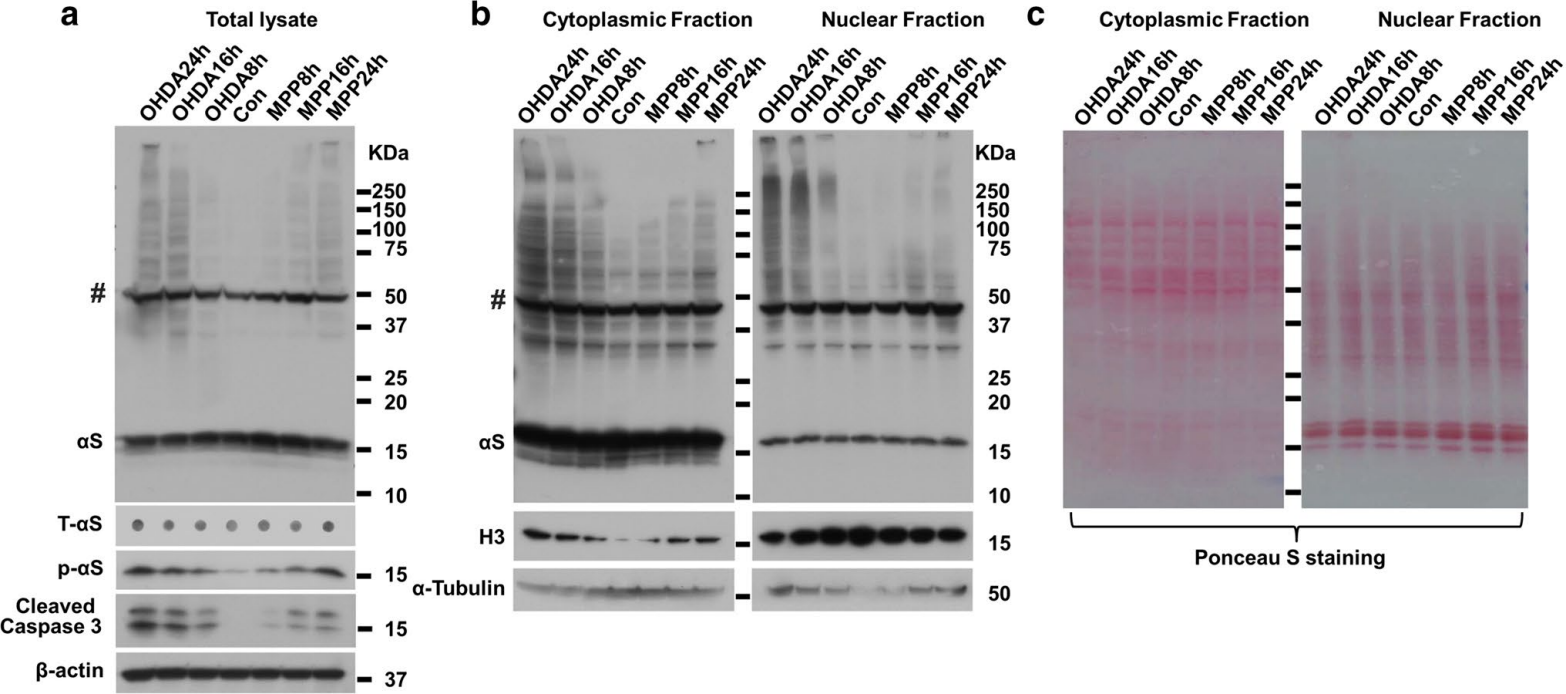
Aggregation and phosphorylation of αS as well as the distribution of αS aggregates in neuronal cells are induced by neurotoxins MPP+ and 6OHDA. Differentiated BE(2)-M17D/3D5 cells with 5 days of αS induction were, respectively, exposed to MPP+ or 6-6OHDA for 8, 16 and 24 h before harvest. Cells without treatment were used as control (Con). Each group of cells was divided into two parts for extraction of total cell lysates or isolation of nuclear and cytoplasm fractions. Samples were subjected to SDS-PAGE followed by western blotting with antibodies to proteins of interest. Dot blots of cell lysates were used to detect the total αS level (T-αS). Molecular weight standards were included as references. **a** Analysis of total lysates showed that aggregation and Serine 129 phosphorylation of αS gradually increased with progression of apoptosis in neuronal cells treated with MPP+ or 6OHDA. **b** Fractionation of cytoplasmic and nuclear proteins revealed that dynamic changes in distribution of αS aggregates and other proteins in neuronal cells induced by MPP+ or 6-6OHDA treated were similar to those induced by STS. **c**. Ponceau S staining was performed to demonstrate that cytoplasmic and nuclear fraction have comparable levels of total proteins

Because STS is not a toxin that triggers PD-related pathology in humans and animal models, we performed similar experiments in neuronal cultures exposed to other neurotoxins commonly used in PD research. Differentiated BE(2)-M17D/3D5 cells overexpressing αS were treated with 1-methyl-4-phenylpyridinium (MPP+) [36, 37] or 6-hydroxy-dopamine (6OHDA) [38, 39]. As expected, time-dependent aggregation and phosphorylation of αS were observed in neuron cultures with apoptosis induced by both neurotoxins (Fig. 4a). Furthermore, nuclear and cytoplasmic fractionation demonstrated that (1) cytoplasmic fractions contained more monomeric αS than nuclear fractions (Fig. 4b); (2) αS aggregates that formed in nuclear fractions had higher molecular weight than those in cytoplasmic fractions; and (3) αS aggregates increased in a time-dependent manner upon induction of apoptosis in both nuclear and cytoplasmic fractions. The changes observed in αS aggregation and phosphorylation and subcellular distribution of αS aggregates in BE(2)-M17D/3D5 cells treated with MPP+ and 6OHDA were similar to those observed with STS, suggesting apoptosis may play a role in toxicity of other neurotoxins, at least in terms of their effects on αS aggregation in neuronal cultures.

### Apoptotic bodies associated with disruption of nuclear membrane integrity contain αS aggregates

**Fig. 5:**
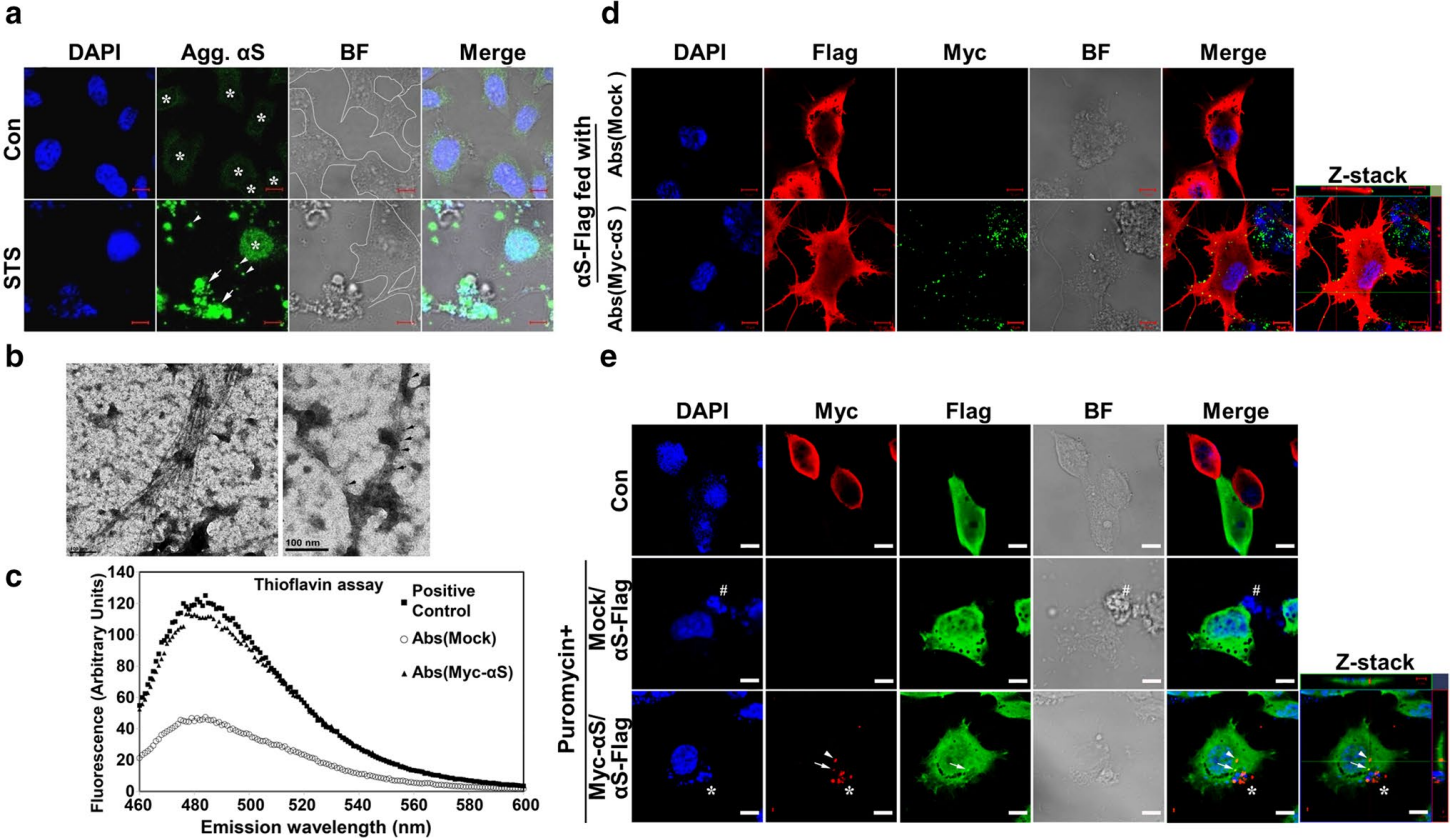
Apoptotic bodies derived from neuronal cells with αS overexpression contain αS aggregates that are taken up by surrounding cells. **a** Apoptotic bodies with disrupted nuclear envelope have αS aggregates. Differentiated BE(2)-M17D/3D5 cells with 5 days of αS expression were induced to enter late apoptotic stage by STS treatment for 36 h and then subjected to immunocytochemical staining with LB509 antibody and nuclear counterstaining with DAPI. Cells with intact nuclei are outlined (*white line*) in bright-field (BF) microscopy image. Signals of aggregated αS (Agg. αS) in cells without STS treatment (Con) are enhanced to demonstrate the cell morphology since the laser setting used for imaging STS-treated cells was not visible in control cells. *Arrows* and *arrowheads* denote apoptotic bodies and other smaller structures. The *asterisk* (*) labeled the location of intact nuclei in both groups. **b** Ultrastructural evaluation of sarkosyl-insoluble fractions prepared from apoptotic bodies. Apoptotic bodies were isolated from differentiated BE(2)-M17D/3D5 cells with 5 days of αS expression and 36 h of STS treatment and then subjected to fractionation to obtain Sarkosyl-insoluble fraction. (*Left*) Sarkosyl-insoluble fraction adsorbed on EM grids and negatively stained with 5 % uranyl acetate revealed filamentous assemblies of diameter about 8–10 nm. (*Right*) The filaments were decorated with 5 nm *gold* particles (*arrows*) by immunogold labeling using an anti-αS antibody, thus verifying that they were assembled from αS. Similar filamentous structures were not detected in apoptotic bodies from null M17D cells (data not shown). *Scale bar*: 100 nm. **c** Thioflavin T binding assay for sonicated apoptotic bodies derived from differentiated Myc-αS cells and mock-transfected cells with 36 h of puromycin treatment to induce apoptosis. Apoptotic bodies containing αS-positive filaments shown in (**b**) were used as positive control. The PBS solution was used as blank. **d** Immunocytochemistry showed Myc immunoreactivity outside and inside αS-Flag cells treated with sonicated apoptotic bodies from Myc-αS cells. Similar findings are not observed in control cells treated with sonicated apoptotic bodies from null M17D cells. Z-stack imaging confirmed the internalization of Myc-positive αS aggregates. **e** Neuronal cells can take up αS aggregates released from surrounding apoptotic cells. αS-Flag cells were co-cultured with Myc-αS or mock transfected M17D cells at equal density and subjected to differentiation for 5 days and then maintained in media with or without puromycin for another 5 days. After that, cells were fixed for immunocytochemical staining with antibodies to Myc and Flag and counterstaining with DAPI. *Arrows* and *arrowheads* denote Myc-positive aggregates from apoptotic bodies. *Asterisk* (*) and *number sign* (*#*), respectively, marked the apoptotic bodies from Myc-αS or mock transfected cells. *Scale bar*: 10 µm for all pictures

Because αS aggregates increasingly formed during apoptosis and because αS aggregation at later stages of apoptosis was associated with unresolvable high-molecular-weight molecular species at the top of gel (Fig. 1b, c), we examined the morphology of αS aggregates in cultures at late stages of apoptotic cell death. Thirty-six hours after STS treatment, differentiated BE(2)-M17D/3D5 cells were subjected to immunocytochemical staining with LB509 to detect αS aggregates [40]. At this late stage of apoptosis, cells showed disrupted nuclear profiles and more αS aggregation than that observed in intact nuclei at earlier stages of apoptosis (Fig. 5a). Of note, αS aggregates were observed not only in apoptotic bodies, but also in smaller structures (arrowheads in Fig. 5a). These findings suggest that a range of αS aggregates can be released into the extracellular milieu from apoptotic cells.

### Apoptotic bodies contain filamentous αS aggregates

Given the striking degree of αS immunoreactivity in apoptotic bodies, we explored their ultrastructural characteristics to determine if apoptosis-associated αS was filamentous, as in Lewy bodies. Cell lysates from differentiated BE(2)-M17D/3D5 cells without STS treatment, and apoptotic bodies isolated from STS-treated differentiated BE(2)-M17D/3D5 cells or null M17D cells, were fractionated. Sarkosyl-insoluble fractions were processed for immunoelectron microscopy. Interestingly, filamentous structures were detected in BE(2)-M17D/3D5 cells derived from apoptotic bodies (Fig. 5b, left), but not in other samples (data not shown). The filaments were immunopositive for αS (Fig. 5b, right), which suggests that filamentous αS can be formed in apoptotic bodies of cells overexpressing αS.

### αS aggregates from apoptotic bodies can be taken up by neuronal cells

Since αS aggregates in apoptotic bodies can be released upon disruption of the cell membrane, we investigated if aggregates of αS can be internalized by surrounding cells as described in previous study [11]. To address this issue, we employed two different neuronal cell models derived from M17D neuroblastoma cell line. One overexpresses Myc-tagged αS (Myc-αS) is neomycin resistant and sensitive to puromycin; the other overexpresses Flag-tagged αS and is puromycin resistant, but sensitive to neomycin. Differentiated Myc-αS cells were treated with puromycin, which triggers apoptosis, for 36 h to produce Myc-αS aggregates associated with apoptotic bodies [41]. Isolated apoptotic bodies were sonicated for a thioflavin T binding assay [30] to first confirm the formation of filamentous αS (Fig. 5c), then added to differentiated αS-Flag cells. Apoptotic bodies from puromycin-treated differentiated mock transfectants were used as a control. After 3 days of exposure to sonicated apoptotic bodies, cells were fixed and immunolabeled with antibodies to Flag and Myc tags. Myc-αS aggregates were observed on the cell surface and in the cytoplasm of most αS-Flag cells treated with Myc-αS sonicated apoptotic bodies, but not in control cells (Fig. 5d). These findings suggest that Myc-αS aggregates can bind to and be taken up by αS-Flag cells.

To determine if neuronal cells can directly take up αS aggregates released from surrounding apoptotic cells, αS-Flag cells were co-cultured with Myc-αS or mock transfected M17D cells at equal density and subjected to differentiation for 5 days and maintained in media with or without puromycin for another 5 days before fixation and immunostaining. Apoptotic bodies, characterized by DAPI-positive nuclear debris, were observed in puromycin-sensitive Myc-αS cells bound to Flag-αS cells (Fig. 5e). In addition, apoptotic bodies in the process of decomposition appeared to be internalized into puromycin-resistant αS-Flag cells, suggesting that the apoptosis-induced αS aggregates can spread to surrounding cells in vitro. Furthermore, about 10 ± 3 % of the αS-Flag cells had internalized Myc-immunopositive particles that were co-labeled with Flag antibody (Fig. 5e), consistent with recruitment of endogenous αS-Flag protein into Myc-αS seeds.

### Apoptosis-derived αS aggregates can be taken up by cells in vivo

**Fig. 6:**
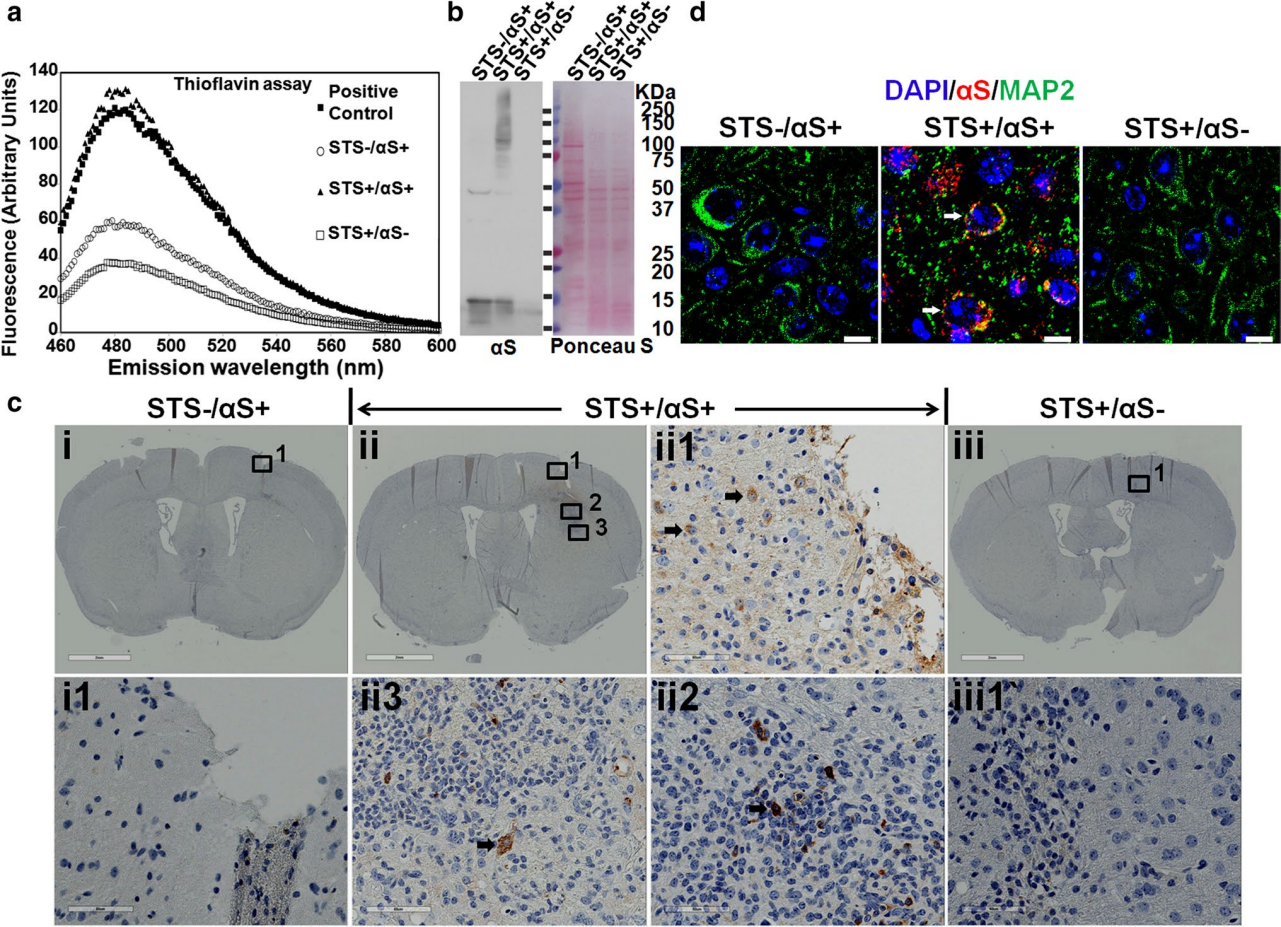
Apoptotic bodies derived αS aggregates can be taken up by neuronal cells in vivo upon inoculation into mouse brain. Apoptotic bodies from STS-treated BE(2)-M17D/3D5 cells with or without αS induction, respectively, referred to as STS+/αS+ and STS+/αS−, were isolated and injected into brains of mice around age of 12 months. Cell lysates from BE(2)-M17D/3D5 cells with αS induction but without STS treatment was also included as another control (STS-/αS+). All injected materials were subjected to **a** thioflavin T assay to confirm the formation of filamentous αS and with SDS-PAGE/western blotting to confirm SDS-insolubility of αS aggregates. Apoptotic bodies containing αS-positive filaments shown in Fig. 5b were used as positive control for (**a**). Sections from the injected brain region were subjected to (**c**) immunohistochemical staining with LB509 to human αS and (**d**) immunofluorescence staining with LB509 and MAP2 to confirm that neuronal uptake of human αS aggregates can be only observed in mouse brain injected with apoptotic bodies (STS+/αS+). *Black arrows* in (**c**) denote neuron-like cells with internalized αS aggregates. *White arrows* in (**d**) denote MAP2 and human αS aggregates positive neurons. *Scale bar*: 2 mm for *i*, *ii* and *iii*, 60 µm for *i1*, *ii1* to *ii3* and *iii1* in (**c**), 10 µm for (**d**)

To determine if apoptosis-induced αS aggregates can be taken up by neighboring cells in vivo, apoptotic bodies from STS-treated differentiated BE(2)-M17D/3D5 cells with and without αS overexpression were isolated and stereotaxically injected into somatosensory cortex and dorsal neostriatum of wild-type C57BL/6 mice at ≈12 months of age following methods of Luk et al. [42]. Cell lysates from BE(2)-M17D/3D5 cells with αS overexpression, but without STS treatment, were included as a control. All injected materials were subjected to thioflavin T assay and SDS-PAGE/western blots (Fig. 6a, b) to confirm the presence of αS aggregates in the materials used for brain injection. After 1 week, brains were harvested, fixed and embedded in paraffin for immunohistochemical studies. Human αS pathology was detected in the vicinity of the injected lysates from STS-treated cells in the form of neocortical neuronal perinuclear αS deposits (Fig. 6d, ii1 in  c), while thread-like and dot-like αS deposits were detected in somatosensory cortex and dorsal neostriatum (Fig. 6c, insets). In contrast, cell lysates not treated with STS or those treated with STS, but not overexpressing αS, did not have neuronal cytoplasmic or neuritic αS pathology (Fig. 6c, insets). These results suggest that αS aggregates from apoptotic neurons can be taken up by neurons in vivo.

## Discussion

Our study suggests that neuronal αS aggregation may be triggered in some cases by proaggregant nuclear factor(s) associated with apoptosis. These initial aggregates may then serve as sources for αS pathology in the immediate vicinity of the affected neurons, which may eventually propagate to more distant sites, possibly by cell-to-cell transmission. Although we demonstrated that filamentous αS aggregates form quickly in apoptotic neurons, it remains to be determined whether this process occurs in PD and related disorders.

Apoptosis is thought to play a role in neuronal cell death in PD and related disorders. Specifically, markers of apoptosis have been demonstrated in postmortem brains of PD [14, 15], as well as animal models [16, 17] and cellular models of PD [18, 19]. Moreover, it has been suggested that apoptosis is the most common type of neuronal death in PD and that there is a proapoptotic environment in the substantia nigra in PD [14]. Therefore, the proapoptotic environment and apoptotic neuronal death in PD and related disorders provide support for a role of apoptosis-associated proaggregant factor(s) in αS aggregation.

Effective clearance mechanisms in the brain quickly remove apoptotic neurons and their breakdown products [43]. Under normal circumstance αS aggregates would most likely be cleared and not propagate to adjacent cells. When neuronal death occurs in vulnerable neuronal populations in PD and related disorders, there may be incomplete clearance of breakdown products from apoptotic neurons and subsequent propagation of αS aggregates. Aging is the major risk factor for PD, and it is associated with decline in function of the immune system [44]. Thus, in the aged brain apoptosis-associated αS aggregates may be more prone to propagation.

A caveat of our studies is that they were conducted in a human dopaminergic cell line that inducibly overexpresses αS. This issue is raised because a critical concentration of αS is required to initiate its assembly [33, 45], and the neuronal cultures used in many PD cell models have αS overexpression. The rationale for using models with αS overexpression is that familial PD with multiplication of the αS gene (*SNCA*) have increased expression of wild-type αS [46–49] as well as  severe αS pathology [50]. Even in normal human aging and in older animal brains, αS expression levels have been shown to be increased compared to younger individuals [51–54]. Although it remains unclear what concentration of αS is critical for its aggregation in PD and related disorders, the fact that αS aggregates can be observed in brains of aged animals and neurologically normal humans indicates that on a cell-by-cell basis there is a sufficient concentration of αS to lead to aggregation even in normal individuals.

We propose that rapid formation of αS aggregates in neurons undergoing apoptosis is a consequence of nucleocytoplasmic barrier disruption and the subsequent interaction between cytoplasmic αS and proaggregant nuclear factor(s). Although previous studies have shown that αS can be transported into the nucleus upon induction of oxidative stress either as an N-terminal truncated form [55] or as full length form mediated by importin [56], these mechanisms are probably not relevant to our observations. First, using fluorescent complementation assays that are dependent upon integration of full-length tagged αS molecules to monitor αS oligomerization, we observed nuclear signals upon indication of apoptosis. Second, we did not detect evidence of truncated αS in western blots of nuclear fractions. Thus, it does not appear that truncation of αS is necessary for nuclear translocation in the setting of apoptosis. Third, our results showed that αS aggregation associated with apoptosis is accompanied by degradation of nuclear envelope (Figs. 1, 2). Although we cannot completely exclude the possibility that some αS may be transported into the nucleus by importin during apoptosis, this physiological process of nuclear pore complex transport may be minimal in a cell where there is considerable disruption of the nuclear envelope.

The αS aggregates formed in apoptotic neurons share important features with pathological αS from PD and related disorders, such as filamentous structures, thioflavin binding, serine-129 phosphorylation and the ability to enter neuronal cells, suggesting similarities between αS aggregates formed under in vitro and in vivo conditions.

Although we clearly demonstrated predominant nuclear localization of proaggregant factor(s) and cellular uptake of αS aggregates formed upon disruption of nuclear envelope, we do not know the nature of nuclear proaggregant factor(s) and how αS rapidly forms aggregates upon interaction with these factors. Since it has been shown that the loss of nuclear integrity is accelerated in post-mitotic cells in an age-dependent manner [57], it is possible that chronic nuclear “leakiness” of proaggregant factor(s) might also contribute to accumulation of αS aggregates in brains of aged human [51].

Overall, our study suggests that a source of pathological αS in PD and related disorders might be from apoptotic neurons in which αS rapidly forms aggregates due to disruption of nuclear membrane and exposure of cytosolic αS to proaggregant nuclear factor(s). Accordingly, at least some αS aggregates might be a secondary phenomenon generated during neuronal apoptosis. The primary insult that leads to apoptosis is unknown; however, once triggered, the effects of apoptosis on αS may accelerate a pathological cascade that eventually leads to local and more distant neurodegeneration. While it is important to determine the cause of neuronal apoptosis or leakiness of nuclear membrane that may possibly expose cytosolic αS to nuclear proaggregant factors in selectively vulnerable neuronal populations in PD and related disorders, finding ways to limit the effects of apoptosis on αS aggregation, deposition, local uptake and propagation might significantly impact progression of the disease. The current observations suggest novel processes in the pathogenesis of PD and related disorders and may open the door to innovative treatments.

## Electronic supplementary material

Below is the link to the electronic supplementary material.

Supplementary material 1 (DOCX 360 kb)
