# Supplementary material for: Proaggregant nuclear factor(s) trigger rapid formation of α-synuclein aggregates in apoptotic neurons
Source: Acta Neuropathol. 2016 Feb 2;132:77–91. doi: 10.1007/s00401-016-1542-4 (PMC4911378; doi:10.1007/s00401-016-1542-4)
Supplement: Supplementary file 1 — Supplementary material 1 (DOCX 360 kb) [file 401_2016_1542_MOESM1_ESM.docx]

**Electronic supplementary material:**


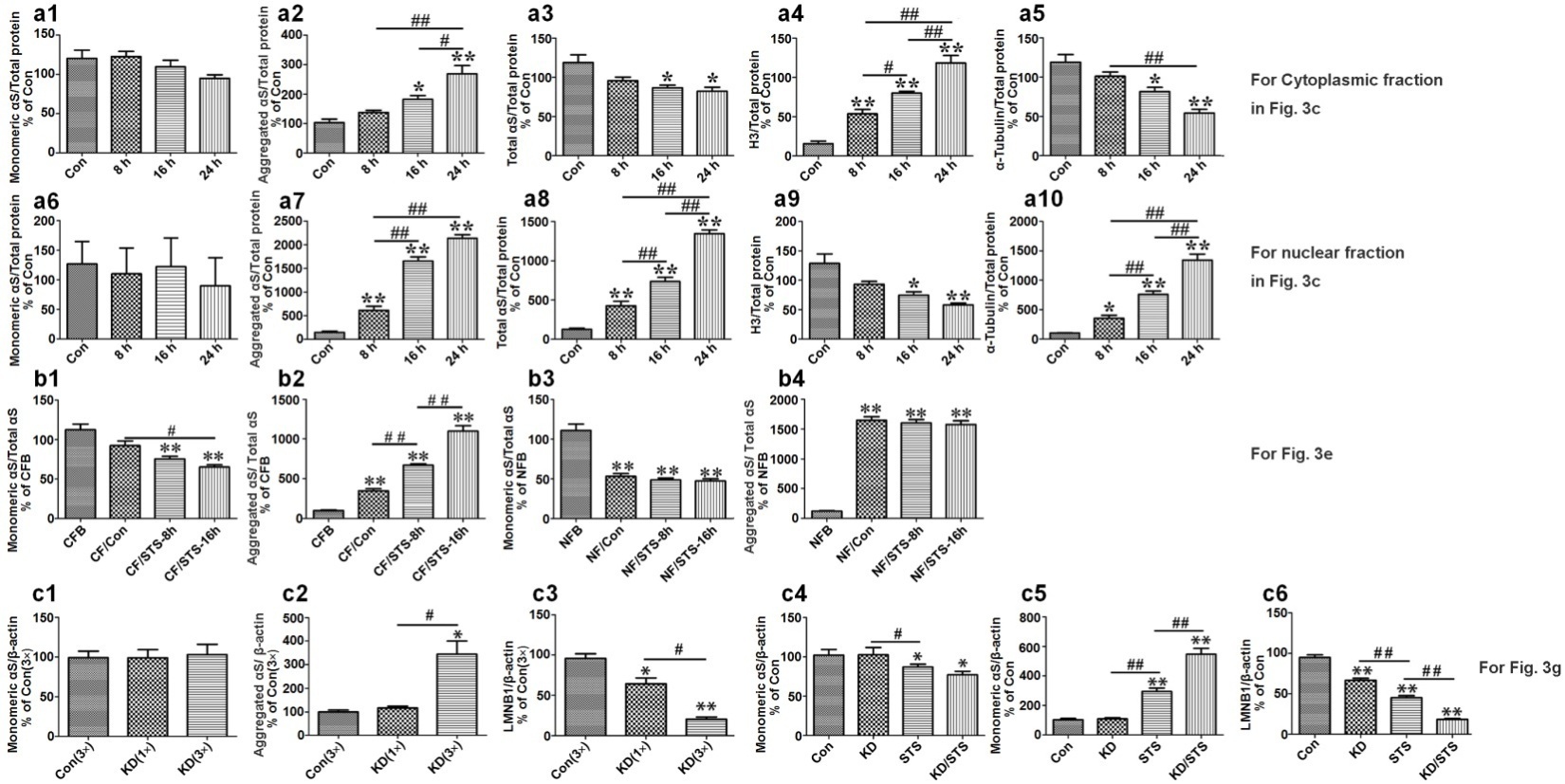


**Figure A1. Statistical analysis of immunoreactivity of various proteins shown in Figure 3.** Bar graphs (**a1 to a5), (a6 to a10**), (**b1 to b4**) and (**c1 to c6**) respectively summarized the results of quantitative analyses of immunoreactivities of various proteins in samples from three independent experiments represented by (3c-cytoplasmic fraction), (3c-nuclear fraction), (3e) and (3g) in Fig. 3 with normalization against β-actin or T-αS immunoreactivities, or densitometric measurement of Ponceau S staining of total protein in each sample. The average values of control group (Con, CFB or NFB) were set as 100%. Error bars represent standard error of the mean (* p < 0.05, ** p < 0.01, comparing to control group; # p < 0.05, ## p < 0.01, comparing subsets linked by line, n=3).

**Table A1-- Quantitation of immunoreactivities of proteins in Fig.4a with normalization against β-actin or T-αS**

|  | | OHDA24h | OHDA16h | OHDA8h | Con | MPP8h | MPP16h | MPP24h |
| --- | --- | --- | --- | --- | --- | --- | --- | --- |
| **Total lysate** | Oligomeric αS | 5.38 | 4.68 | 1.88 | 1.00 | 1.47 | 2.35 | 2.91 |
|  | Monomeric αS | 0.93 | 0.94 | 0.94 | 1.00 | 1.07 | 1.05 | 0.95 |
|  | p-αS | 6.44 | 5.07 | 3.55 | 1.00 | 2.05 | 3.79 | 5.57 |
|  | Cleaved Caspase 3 | 33.66 | 25.96 | 13.84 | 1.00 | 3.42 | 9.78 | 12.46 |

**Table A2—Quantitation of immunoreactivities of proteins in Fig.4b with normalization against densitometric measurement of Ponceau S staining in Fig. 4c**

|  | | OHDA  24h | OHDA  16h | OHDA  8h | Con | MPP  8h | MPP  16h | MPP  24h |
| --- | --- | --- | --- | --- | --- | --- | --- | --- |
| CF | Oligomeric αS | 3.09 | 2.46 | 1.72 | 1.00 | 1.12 | 1.28 | 1.70 |
|  | Monomeric αS | 0.97 | 0.98 | 0.98 | 1.00 | 0.97 | 0.97 | 0.99 |
|  | H3 | 14.62 | 10.93 | 6.35 | 1.00 | 3.04 | 7.68 | 10.29 |
|  | α-Tubulin | 0.43 | 0.54 | 0.74 | 1.00 | 0.96 | 0.80 | 0.51 |
| NF | Oligomeric αS | 3.51 | 2.93 | 1.63 | 1.00 | 1.23 | 1.50 | 1.69 |
|  | Monomeric αS | 0.99 | 1.00 | 1.00 | 1.00 | 0.99 | 0.98 | 0.98 |
|  | H3 | 0.54 | 0.81 | 0.94 | 1.00 | 0.88 | 0.79 | 0.65 |
|  | α-Tubulin | 12.06 | 5.99 | 4.58 | 1.00 | 1.91 | 5.10 | 7.58 |

CF- Cytoplasmic Fraction; NF- Nuclear Fraction.
